# Steric and Energetic Studies on the Synergetic Enhancement Effect of Integrated Polyaniline on the Adsorption Properties of Toxic Basic and Acidic Dyes by Polyaniline/Zeolite-A Composite

**DOI:** 10.3390/molecules28207168

**Published:** 2023-10-19

**Authors:** Ayah T. Zaidalkilani, Amna M. Farhan, Islam R. Sayed, Ahmed M. El-Sherbeeny, Wail Al Zoubi, Ammar Al-Farga, Mostafa R. Abukhadra

**Affiliations:** 1 Department of Nutrition, Faculty of Pharmacy and Medical Sciences, University of Petra, Amman 11196, Jordan; 2 Materials Technologies and Their Applications Lab, Geology Department, Faculty of Science, Beni-Suef University, Beni-Suef 65211, Egypt; 3 Chemistry Department, Faculty of Science, Beni-Suef University, Beni-Suef 65211, Egypt; 4 Geology Department, Faculty of Science, Beni-Suef University, Beni-Suef 65211, Egypt; 5 Industrial Engineering Department, College of Engineering, King Saud University, P.O. Box 800, Riyadh 11421, Saudi Arabia; 6 Materials Electrochemistry Laboratory, School of Materials Science and Engineering, Yeungnam University, Gyeongsan 38541, Republic of Korea; 7 State Key Laboratory of Food Science and Technology, Jiangnan University, Wuxi 214122, China

**Keywords:** zeolite-A, polyaniline, synthetic dyes, equilibrium, steric, energetic

## Abstract

The synergetic enhancement effect of the polyaniline (PANI) integration process on the adsorption properties of the PANI/zeolite-A composite (PANI/ZA) as an adsorbent for malachite green and Congo red synthetic dyes was evaluated based on classic equilibrium modelling in addition to the steric and energetic parameters of advanced isotherm studies. The PANI/ZA composite displays enhanced adsorption capacities for both methylene blue (270.9 mg/g) and Congo red (235.5 mg/g) as compared to ZA particles (methylene blue (179.6 mg/g) and Congo red (140.3 mg/g)). The reported enhancement was illustrated based on the steric parameters of active site density (Nm) and the number of adsorbed dyes per active site (n). The integration of PANI strongly induced the quantities of the existing active sites that have enhanced affinities towards both methylene blue (109.2 mg/g) and Congo red (92.9 mg/g) as compared to the present sites on the surface of ZA. Every site on the surface of PANI/ZA can adsorb about four methylene blue molecules and five Congo red molecules, signifying the vertical orientation of their adsorbed ions and their uptake by multi-molecular mechanisms. The energetic investigation of the methylene blue (−10.26 to −16.8 kJ/mol) and Congo red (−9.38 to −16.49 kJ/mol) adsorption reactions by PANI/ZA suggested the operation of physical mechanisms during their uptake by PANI/ZA. These mechanisms might involve van der Waals forces, dipole bonding forces, and hydrogen bonding (<30 kJ/mol). The evaluated thermodynamic functions, including enthalpy, internal energy, and entropy, validate the exothermic and spontaneous behaviours of the methylene blue and Congo red uptake processes by PANI/ZA.

## 1. Introduction

One of the most important issues affecting mankind is water contamination, which becomes worse as industrial activity rises [1]. The contaminants produced by these industries included several compounds such as dyes, insecticides, medicines, heavy metals, chemical ions, microplastics, nitrate, and sulphate [2]. Approximately 7 × 10^5^ tons of hazardous synthetic dyes are generated yearly from frequently released organic pollutants, with more than 20% of these compounds being released into the natural environment as wastewater [3]. Different species of synthetic dyes have been introduced into industrial societies as key aromatic compounds for various uses. However, the majority of frequently used dyes have chemical characteristics that are both poisonous and very resistant to biodegradation [4,5]. Environmentally, the presence of these types of synthetic organic compounds in water systems significantly impairs both the bioactivity and photosynthetic activity of aquatic species by obstructing the effectiveness of sunlight’s penetration [6,7].

Among the synthetic dyes, methylene blue (MB) is a known type of basic azo dye that has a complicated aromatic structure (C_16_H_18_ClN_3_S) and is extensively used in some pharmaceutical and cosmetics applications in addition to different industries, such as textile, printing, food, paper, plastic, and photography [8,9]. However, the detection of it as a dissolved compound in water resources at unacceptable concentrations is associated with numerous health and environmental side effects. This includes several diseases such as breathing diseases, nausea, cancer, convulsions, hypertension, vomiting, renal problems, and damage to the reproductive system [10,11,12]. Regarding its environmental applications, the MB contaminants cause significant depletion in the oxygen content in the aquatic environment and negatively affect the photosynthesis activities of aquatic organisms by reducing the light penetration efficiency [13,14]. In addition, Congo red (CR) is a common type of acidic azo dye (C_32_H_22_N_6_Na_2_O_6_S_2_; amino-1-aminonaphthalene sulphonic)that is widely used in printing, papers, printing, leather, the polymer industry, textile dyeing, and rubber industries. CR was also detected as a pollutant in water supplies, causing some health side effects such as irritation to the skin, eyes, respiratory system, and reproductive system [3,15]. The high biodegradable resistance of CR dye and the carcinogenic and mutagenic properties of its altered benzidine make its existence in water resources have strong negative impacts on human health (nausea, allergies, vomiting, irritation of the digestive system, and diarrhoea) as well as aquatic life [11,16,17].

Based on previous environmental and health considerations, numerous techniques have been developed to get rid of these dyes, including photocatalytic reactions [18], ozonation [19], flocculation/coagulation [20], co-precipitation [21], ion exchange [22], and adsorption [23]. Its low operation and production costs, high removal capacity, large-scale applicability, significant recyclability, and facile production methods strongly recommended the adsorption removal of dyes as an effective decontamination method for MB as well as CR [24,25]. Therefore, numerous materials of single and hybrid phases were studied as potential adsorbents for dyes, including graphene oxide/CuFe_2_O_4_ [1], several polyacrylonitrile (PAN) compounds [26], chitosan/goethite [27], moringa oleifera/polyacrylonitrile [17], ZrO_2_/MgAl-LDH [28], zinc curcumin oxide nanoparticles [8], amorphous iron oxide nanoparticles [29], CuB_2_O_4_ [30], biopolymer/graphene oxide [31], and zeolite [32]. However, the key parameters for selecting the best adsorbents are their natural availability, fabrication cost, fast kinetic rates, adsorption efficiency, adsorption selectivity, and reusability value [33].

Later, heterogeneous and multifunctional structures with various organic/inorganic reactive chemical groups based on natural precursors were assessed as improved potential adsorbents during the decontamination of organic molecules [34,35]. Currently, synthesized zeolite/biopolymers that establish excellent adsorption capacity, recycling potential, biodegradable properties, ion exchange capacity, and environmental advantages are presented as prospective multifunctional and low-cost adsorbents [36,37]. The synthesized forms of zeolite have been described as superior materials with a wide range of physicochemical advantages and may be combined effectively with various forms of constituents to develop a hybrid structure with improved physical and chemical characteristics [38,39,40]. Synthetic zeolite-A has noteworthy structural flexibility, chemically stable properties, surface area, remarkable non-toxicity, adsorption efficiency, and excellent biocompatibility [36,41]. The key drawback of these types of materials is their significant hydrophilicity, which adversely affects their affinity and reactivity towards the organic compounds that exist in water [42]. This may be prevented by the innovative functionalization of the surface of zeolite with various species of polymeric chains and surfactants that can improve the organophilicity and reactivity of their surfaces, as well as the distribution of their structural pore sizes and the determined surface area [43,44].

Among the commonly used polymers that exhibit attractive adsorption properties, polyaniline polymer was studied as an effective adsorbent, either as a single phase or as an integrated component in organic/organic or inorganic/organic composites. Technically, it is characterized by its conductive properties, low preparation cost, high surface area, non-toxic properties, significant thermal stability, and high adsorption capacity [45,46,47]. It was expected that the functionalization of synthetic zeolite-A with polyaniline chains would result in an innovative hybrid structure with enhanced adsorption capacity and numerous active adsorption sites for chemical groups to be applied in a more effective decontamination of organic dyes as compared to the synthetic zeolite as a single component. The present study involved the synthesis and characterization of a polyaniline/zeolite-A composite as an enhanced adsorbent for both acidic CR dye and basic MB dye in aqueous solutions. This involved a deep investigation of the polymer integration effects on the adsorption capacity, the textural properties, the number of active sites, the energetic properties, and the adsorption mechanism, considering the commonly studied classic kinetic and isotherm models in addition to the advanced equilibrium studies based on the statistical physics theory and the associated steric and energetic parameters.

## 2. Results and Discussion

### 2.1. Effect of the pH

**Figure 1:**
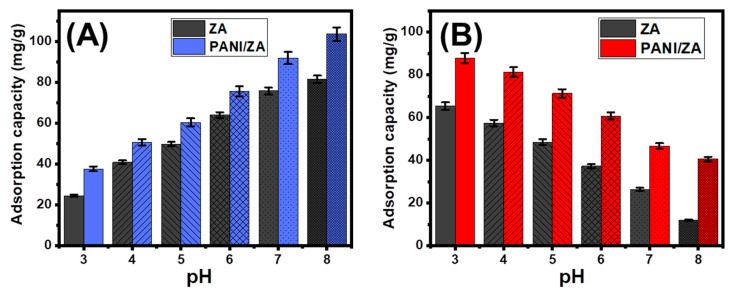
The impact of the solutions pH on the adsorption of MB dye (**A**) and CR dye (**B**) by both ZA and PANI/ZA composite.

The pH parameter is an important part of the adsorption process because it affects the surface charge of the adsorbent and the ionization behaviour of the dissolved pollutants in the aqueous solutions. The adsorption behaviours of ZA and PANI/ZA in the removal of the CR and MB dyes were investigated, including a pH range of 3 to 8, and the other parameters were fixed at the following constant values: a volume of 100 mL, a concentration of 100 mg/L, a dosage of 0.4 g/L, and a test temperature of 20 °C. Based on the amounts of CR and MB adsorbed using both ZA and PANI/ZA, there was a clear improvement in the removal of MB and a significant declination in the uptake of CR when the pH of the polluted solutions tested increased from a pH of 2 (Figure 1A,B). The MB uptake capacities increased from 24.4 mg/g (ZA) and 37.6 mg/g (PANI/ZA) at a pH of 2 up to 81.4 mg/g (ZA) and 103.6 mg/g (PANI/ZA) at a pH of 8 (Figure 1A). For the CR dye, the uptake properties declined from 65.3 mg/g (ZA) and 87.3 mg/g (PANI/ZA) at a pH of 2 down to 11.9 mg/g (ZA) and 40.5 mg/g (PANI/ZA) at a pH of 8 (Figure 1B). 

Therefore, the evaluated structures will be qualified to serve as highly effective adsorbents during the actual remediation activities of MB dye and considerable activity during the removal of CR in accordance with the pH level of industrial wastewater set by the US EPA (pH 6 to 9) and the experimental behaviours of PANI/ZA at various pH values [48]. Both the ionization characteristics of MB and CR dyes and the dominant surficial charges on the structures of ZA as well as PANI/ZA can be employed to demonstrate the reported differences in the uptake properties. In terms of the MB ionization characteristics, the positively charged ions of basic MB ions display high electrostatic attraction characteristics with the previously established negatively charged structural groups of ZA and PANI/ZA at high pH levels (alkaline circumstances). The contrast can be reported for the acidic ions of CR that exhibit strong repulsive properties with these functional groups at basic conditions and remarkable attractive forces with these groups during their protonation states at acid conditions [48,49,50,51].

### 2.2. Kinetic Studies

#### 2.2.1. Effect of Contact Time

**Figure 2:**
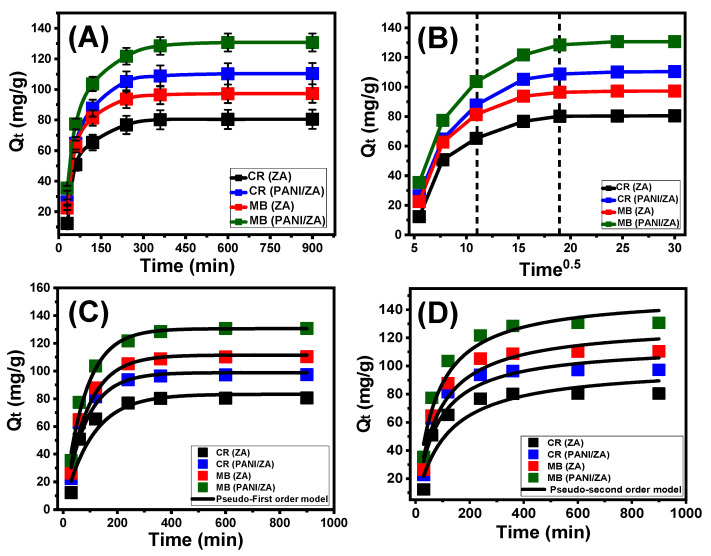
The impact of the contact time on the adsorption of MB and CR dyes by both ZA and PANI/ZA (**A**), the intra-particle diffusion curves for the uptake of MB and CR by ZA and PANI/ZA (**B**), the fitting of the MB and CR adsorption results with pseudo-first order kinetic model (**C**), and fitting of the MB and CR adsorption results with pseudo-second order kinetic model (**D**).

The experimental effect of the adsorption period on the MB and CR decolourization capacities accomplished by ZA and PANI/ZA have been determined within an assessed range of 30 min to 900 min at adjusted levels of the principal influencing factors (concentration: 100 mg/L; dosage: 0.4 g/L; volume: 100 mL; temperature: 20 °C; pH: 8 for MB and 3 for CR). The decolourization characteristics of ZA as well as its composite (PANI/ZA) as possible MB and CR adsorbents justify the noticeably increased rates in addition to the adsorbed amounts in mg/g and the controllable extension of the testes period (Figure 2A). This improvement’s effects may really be noticed after 360 min, either using ZA or PANI/ZA. After that, the prolonged duration of the experiments shows no appreciable effects in terms of the uptake rates or the amounts of sequestered MB or CR, revealing stabilization or equilibrium situations (Figure 2A). In this state, the ZA and PANI/ZA particles display the equilibrium levels of MB (97.3 mg/g (ZA) and 130.7 mg/g (PANI/ZA)) and CR (80.5 mg/g (ZA) and 110.4 mg/g (PANI/ZA)) dye adsorption capacities (Figure 2A). The rapid rates and sudden rise in the amount of adsorbed MB and CR were caused by an abundance of massive quantities of unoccupied active sites of the ZA and PANI/ZA particles during the beginning periods of the decolourization reactions [36]. As the testing time expands, more and more MB and CR dyes are absorbed into the ZA and PANI/ZA free sites, causing these sites to become occupied and consumed and drastically reducing their availability. As a result, the experimental rates of adsorption significantly decreased over a certain period of time, and the ZA and PANI/ZA particles exhibited ignored improvements in their adsorption characteristics. After completely occupying all of the already available sites with both MB and CR, the equilibrium state of ZA and PANI/ZA were identified [52].

#### 2.2.2. Intra-Particle Diffusion Behaviour

The MB and CR adsorption processes carried out using ZA and PANI/ZA display an intra-particle diffusion curve with segmentation characteristics and show three distinct phases lacking intersection with the initial points of the curve (Figure 2B). This shows that both MB and CR are taken up by collaborative processes in addition to the substantial role played by the ion diffusion of them towards the active receptors of ZA and PANI/ZA [53,54]. This might entail (A) adsorption by active sites along the exterior surface (border), (B) intra-particle diffusion, and (C) the action of the saturating or equilibrium stage [55]. The first stage indicates that the exterior adsorption mechanisms of MB as well as CR were active when the tests initially started, and the effectiveness of this stage depends on the total amount of surface-active receptors (Figure 2B) [56]. A new stage that denotes the performance of additional mechanisms, including the impact of both the MB and CR diffusion activities and what are known as layered adsorption reactions, was identified by extending the time (Figure 2B) [36,55]. Finally, it was identified that the third stage predominates whenever the ZA and PANI/ZA particles are in the equilibrium condition for the MB and CR adsorption reactions. This confirms that the captured MB and CR ions have occupied or consumed the efficient binding sites completely (Figure 2B) [54]. The MB and CR uptake activities at this phase are regulated by a variety of mechanisms, some of which may include molecular interaction and/or mechanisms including interionic attraction [57].

#### 2.2.3. Kinetic Modelling

The established kinetic hypotheses of the pseudo-first order (P.F.) and pseudo-second order (P.S.) models were employed for displaying the kinetic characteristics of the MB and CR processes of adsorption by ZA and PANI/ZA particles. The concordance between the kinetic hypotheses of the two evaluated models and the observed MB and CR uptake behaviours was assessed using the established non-linear fitting levels with their corresponding equations depending on both correlation coefficient (R^2^) and Chi-squared (χ^2^) (Table 1; Figure 2C,D). The acquired values of R^2^ and χ^2^ indicated a better match of the MB and CR processes of adsorption by ZA and PANI/ZA particles with the kinetic characteristics and fundamentals of the P.F. model versus the evaluated P.S. model. The striking concordance between the experimentally detected capacities of ZA and PANI/ZA at the equilibrium states during the uptake of MB (97.3 mg/g (ZA) and 130.7 mg/g (PANI/ZA)) and CR (80.5 mg/g (ZA) and 110.4 mg/g (PANI/ZA)) and the theoretically calculated values (MB (111.5 mg/g (ZA) and 130.55 mg/g (PANI/ZA)) and CR (83.2 mg/g (ZA) and 112.8 mg/g (PANI/ZA))) corroborated these reasonable findings (Table 1). In accordance with the kinetic fundamentals of the P.F. model, the captures of MB and CR by ZA and PANI/ZA particles were primarily caused by physical processes, which may have included notable effects from van der Waals forces and/or electrostatic attraction [58,59]. Even if the P.F. model’s equation for decontaminating MB and CR via ZA and PANI/ZA particles is better suited than the P.S. model’s represented equation, the uptake activities still show a significant match with the P.S. kinetics. Thus, it was anticipated that several common weak chemical mechanisms, such as hydrogen binding, chemical complexing, electron sharing, and hydrophobic interaction, would have a supporting role or have a minimal influence on the reactions [54,58]. The combination of both chemical and physical processes entailed the production of chemically uptaken MB and CR layers, which were then followed by the development of a physically uptaken layer utilizing the initial one as the base [59].

### 2.3. Equilibrium Studies

#### 2.3.1. Effect of MB and CR Concentrations

**Figure 3:**
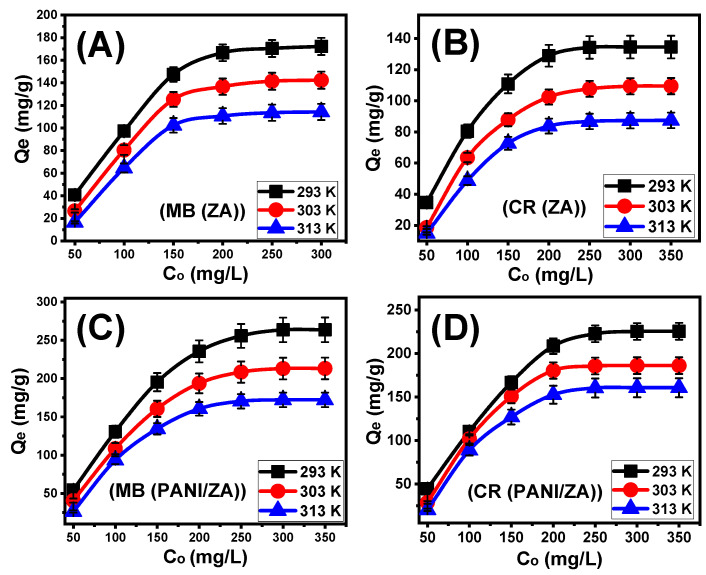
The impact of the starting concentrations on the adsorption of MB dye by ZA (**A**), adsorption of CR dye by ZA (**B**), adsorption of MB dye by PANI/ZA composite (**C**), and adsorption of CR dye by PANI/ZA composite (**D**).

After carefully adjusting all of the influencing factors to specific values (time: 24 h; volume: 100 mL; solid dosage: 0.4 g/L; temperature: (293 K, 303 K, and 313 K); and pH: 8 for MB and 3 for CR), the effect of the starting investigated MB and CR concentrations on the adsorption characteristics of ZA and PANI/ZA was determined throughout an experimental range from 50 to 35 mg/L for CR and up to 300 mg/L for MB. During the evaluation of the adsorption characteristics of ZA and PANI/ZA, the starting concentration is a powerful variable for describing equilibrium features and maximal capacities (Figure 3A–D). After examining the characteristics of ZA and PANI/ZA in the presence of higher levels of MB and CR ions, the amount of MB and CR that were adsorbed substantially rose (Figure 3A–D). The elevated levels of MB and CR in specific volumes trigger a substantial increase in both the diffusion, transportation, and driving forces of the dye ions, resulting in collisions and chemical interactions with the spatially spread active sites on the exterior of ZA and PANI/ZA and, as a result, an improvement in uptake effectiveness [54,57]. The rise in the MB and CR adsorbed amounts in relation to the beginning investigated concentrations may be clearly realized up to specific examined levels (Figure 3A–D). Following that, a rise in the initial MB and CR concentrations exhibits ignored influences on the adsorbed amounts of their molecules either by ZA or PANI/ZA, which distinguish the equilibrium situation and attend to the real optimal adsorption specifications. After 250 mg/L of MB, the equilibrium of ZA as an adsorbent can be detected with actual capacities of 172.2 mg/g (293 K), 142.3 mg/g (303 K), and 114.2 mg/g (313 K) (Figure 3A), while the reported values for PANI/ZA are 263.8 mg/g (293 K), 213.3 mg/g (303 K), and 172.4 mg/g (313 K) (Figure 3C). Regarding the uptake of CR, the reported equilibrium or maximal capacities of ZA are 134.5 mg/g (293 K), 109.4 mg/g (303 K), and 87.6 mg/g (313 K) (Figure 3B) and the measured values of PANI/ZA are 225.6 mg/g (293 K), 186.1 mg/g (303 K), and 160.7 mg/g (313 K) (Figure 3D). The notable high uptake abilities of PANI/ZA during the elimination of both MB and CR versus those of ZA free particles could be attributed to (1) the increased surface area, (2) the substantial rise in the overall number of active sites after the incorporation of the polyaniline chains, and (3) the significant improvement in the composite’s organophilicity when compared to hydrophilic zeolite. The observed decrease in the adsorption of MB and CR by ZA and PANI/ZA in relation to the test’s temperature indicated that the reactions were exothermic.

#### 2.3.2. Giles’s Classification

The categorization of the MB and CR isotherm curves utilizing ZA and PANI/ZA based on the ascribed criteria of Giles’s classification revealed that they formed L-type equilibrium curves (Figure 3A–D). The L-type equilibrium characteristics contribute to the significant and powerful impacts caused by intermolecular attraction forces throughout the adsorption processes of MB and CR by the ZA and PANI/ZA particles, together with the substantial interaction between the dye molecules and the highly reactive chemical structures of ZA and PANI/ZA particles [59]. A full production of the adsorbed MB and CR monolayers on the outermost surfaces of the ZA and PANI/ZA particles was also potentially predicted based on L-type isotherm characteristics [60]. Furthermore, this isothermal behaviour suggests the formation of the ZA and PANI/ZA particles with numerous types of both active and free uptake receptors that exhibit substantial selectivity to the MB and CR molecules throughout their adsorption, particularly at low starting concentrations.

#### 2.3.3. Classic Isotherm Models

**Figure 4:**
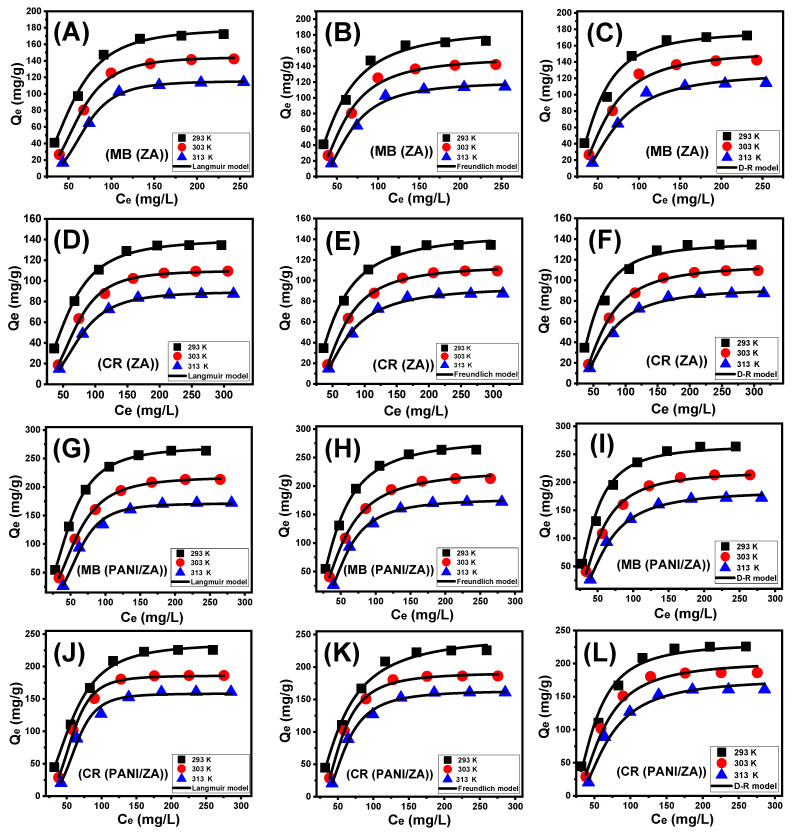
Fitting of the MB adsorption by ZA results with classic isotherm models ((**A**) (Langmuir), (**B**) (Freundlich), and (**C**) (D-R model)), fitting of the CR uptake by ZA results with classic isotherm models ((**D**) (Langmuir), (**E**) (Freundlich), and (**F**) (D-R model)), fitting of the MB adsorption by PANI/ZA composite results with classic isotherm models ((**G**) (Langmuir), (**H**) (Freundlich), and (**I**) (D-R model)), and fitting of the CR uptake by PANI/ZA composite results with classic isotherm models ((**J**) (Langmuir), (**K**) (Freundlich), and (**L**) (D-R model)).

Following the isotherm principles of the standard Langmuir, Freundlich, and Dubinin–Radushkevich (D-R) models, the equilibrium responses of the MB and CR uptake activities by the ZA and PANI/ZA particles were characterized. On the basis of the realized non-linear fits degrees with the corresponding formulas of each model that depended on the values of correlation coefficient (R^2^) alongside Chi-squared (χ^2^), the compatibility between the equilibrium hypotheses of each of these models and the MB and CR uptake behaviours was assessed (Table 2; Figure 4). The values of R^2^ and χ^2^ established slightly a better match of the MB and CR uptake activities by the ZA and PANI/ZA particles with Langmuir isotherm principles (Figure 4; Table 2) rather than the essential characteristics of the Freundlich equilibrium isotherm (Figure 4; Table 2). The Langmuir isotherm assigns MB and CR adsorption homogeneously via the unoccupied and active sites of ZA and PANI/ZA particles and in monolayer orientations [58,59]. Furthermore, the computed equilibrium parameter (RL) values of the MB and CR uptake activities by the ZA and PANI/ZA particles are less than one, indicating that the reactions have favourable properties [56]. The expected maximal MB absorption capacities (Q_max_) of ZA are 179.6 mg/g (293 K), 145.1 mg/g (303 K), and 115.6 mg/g (313 K) and the computed values using PANI/ZA are 270.9 mg/g (293 K), 218.02 mg/g (303 K), and 177.4 mg/g. For CR, the theoretical Q_max_ using ZA are 140.3 mg/g (293 K), 110.04 mg/g (303 K), and 89.6 mg/g (313 K) and for PANI/ZA are 235.5 mg/g (293 K), 189.8 mg/g (303 K), and 166.4 mg/g (Table 2). 

The isotherm principles of the D-R model (Figure 4) describe the energy variation of the ZA and PANI/ZA particles during the capture of the MB and CR dyes, independent of the characteristics of their surfaces, which may be heterogeneous or homogeneous [61]. The significance of Gaussian energy (E) as a theoretical parameter derived from the D-R analysis procedure significantly appoints what type of MB and CR uptake processes influence it (chemical or physical). Adsorption reactions with E values of 8 KJ/mol, 8–16 KJ/mol, and >16 KJ/mol indicate the influence of strongly physical, poor chemical, intricate physical/chemical, and powerful chemical characteristics, respectively [61]. The measured values of the E parameter of the MB and CR uptake by ZA activities are among the estimated energy levels of physical processes (<8 KJ/mol), as well as among the denoted range of zeolitic ion exchange processes (0.6–25 KJ/mol), in accordance with the theoretical results of the kinetic investigations (Table 2). For the PANI/ZA composite, the Gaussian energy for the uptake of CR is within the previously mentioned ranges of physical processes and zeolitic ion exchange mechanisms, while the uptake of MB in low-temperature conditions matches slightly the reported range for complex physical/chemical mechanisms or weak chemical processes. 

#### 2.3.4. Advanced Isotherm Modelling

**Figure 5:**
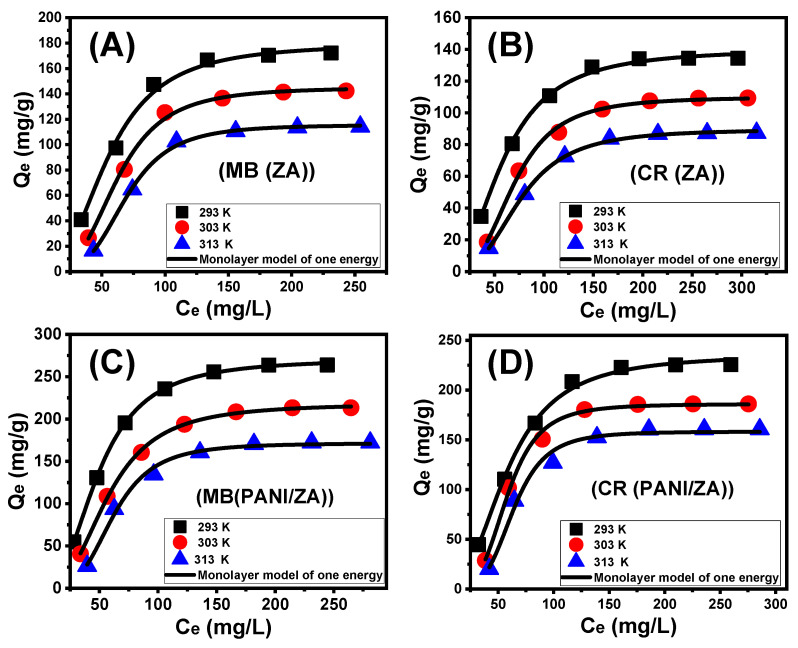
Fitting of the adsorption by ZA (MB dye (**A**) and CR dye (**B**)) and PANI/ZA composite (MB dye (**C**) and CR dye (**D**)) results with advanced monolayer model of one energy.

Depending on the statistical physics theory concept, the latest investigated advanced equilibrium models may significantly indicate the specific characteristics of the adsorption processes with regard to the adsorbent–adsorbate interfaces and the surface characteristics of the incorporated solid adsorbents. The corresponding mathematical factors derived from these models, such as the energetic and steric factors, can potentially be utilized to emphasize the mechanistic procedures. The steric factors included the quantity of occupied functional sites of ZA and PANI/ZA by MB (Nm _(MB)_) and CR (Nm _(CR)_), the total number of MB and CR molecules captured at each site (MB (n _(MB)_) and CR (n _(CR)_), and the uptake capacities of ZA and PANI/ZA at the saturation condition (Q_sat (MB)_ (MB) and Q_sat (CR)_ (CR)). The energetic factors were MB and CR uptake energy (ΔE) in addition to internal energy (E_int_), free enthalpy (ΔG), and entropy (Sa). On the basis of non-linear fits employing this model’ illustrative equations, a simulation of the MG and CR uptake activities has been performed. This has been accomplished using multivariable nonlinear regression modelling and the Levenberg–Marquardt iterative method. A monolayer model with one energy site was employed to depict the MB and CR adsorption processes by ZA and PANI/ZA based on the marked fitting degrees (Table 3; Figure 5A–D).

##### Steric Properties

Number of adsorbed MB and CR (n) per each site

**Figure 6:**
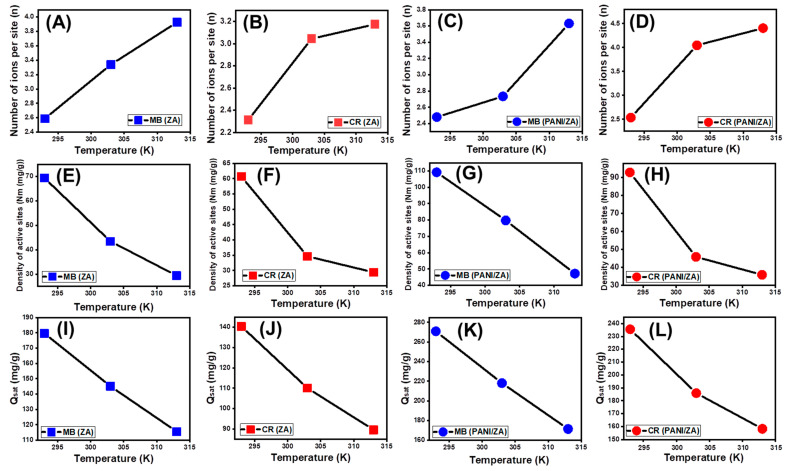
The changes in the numbers of adsorbed dyes molecules by ZA (MB (**A**) and CR (**B**)) and by PANI/ZA (MB (**C**) and CR (**D**)), changes in the active sites density during the uptakes of the dyes by ZA (MB (**E**) and CR (**F**)) and by PANI/ZA (MB (**G**) and CR (**H**)), and changes in the dyes adsorption capacities during the saturation states of ZA (MB (**I**) and CR (**J**)) and PANI/ZA (MB (**K**) and CR (**L**)).

The numerical values of the n _(MB)_ and n _(CR)_ factors substantially suggest the orientation by which the dyes molecules were adsorbed on the surfaces of the ZA and PANI/ZA (vertical or horizontal), as well as their implications for the influencing mechanistic processes (multi-docking versus multi-interactions). The processes that are mostly influenced by multi-anchorage or multi-docking mechanistic processes comprise the capture of one MB or CR molecule by numerous uptake sites in horizontal forms. The retention systems that exhibit value less than one are connected with the horizontal orientation of the adsorbed ions. In contrast, systems with values higher than one display non-parallel as well as vertical orientation for the adsorbed MB or CR, and the occurring uptake activities are primarily influenced by multi-ionic mechanistic processes comprising the capture of a number of dyes per single site [58]. The estimated n levels throughout the capture of MB and CR by ZA (n _(MB)_ = 2.58–3.92; n _(CR)_ = 2.31–3.17) are greater than one (Figure 6A,B; Table 3). These were also reported for the number of adsorbed MB and CR molecules per site on the surface of PANI/ZA (n _(MB)_ = 2.48–3.65; n _(CR)_ = 2.53–4.5) (Figure 6C,D; Table 3). As a result, the MB and CR molecules were taken up via multi-molecular mechanistic processes, whereby each uptake site of the ZA and PANI/ZA could receive up to three ions and be oriented vertically in non-parallel properties. While each site on the surface of ZA can adsorb up to four molecules of both MB and CR, each site on the surface of the PANI/ZA can uptake up to four molecules of MB and up to five molecules of CR, reflecting an enhancement in the affinities of the receptor sites towards the acidic molecules after the integration of polyaniline. 

In the context of the impacts of temperature, the obtained n _(MB)_ and n _(CR)_ values of ZA and PANI/ZA confirm that they arise as a consequence of the elevation in temperature from 293 K to 313 K (Figure 6A–D). This was often attributed to the hypothesized increase in the MB and CR aggregation characteristics during its adsorption by ZA and PANI/ZA at elevated temperatures [47]. This also illustrates the presence of thermal (energetic) activation mechanisms prior to the capture of MB and CR by ZA and PANI/ZA [62,63].

Occupied active sites density (Nm)

The density of the MB- and CR-occupied sites (Nm _(MB)_ and Nm _(CR)_) of ZA and PANI/ZA substantially represents the total quantity of the free and efficient adsorption receptors across the exteriors of its nanoparticles during the reaction (Figure 6E–H; Table 4). ZA has estimated Nm _(MB)_ values of 69.4 mg/g (293 K), 43.5 mg/g (303 K), and 29.45 mg/g (313 K) (Figure 6E; Table 3). These values increased significantly after the compositing of ZE with PANI (PANI/ZA) to 109.2 mg/g (293 K), 79.7 mg/g (303 K), and 47.5 mg/g (313 K) (Figure 6G; Table 3). These were also reported also during the uptake of CR molecules; the estimated occupied site densities by CR (Nm _(CR)_) on the surfaces of ZA are 60.67 mg/g (293 K), 34.66 mg/g (303 K), and 29.43 mg/g (313 K) (Figure 6F; Table 3) while the estimated values using PANI ZA are 92.9 mg/g (293 K), 45.93 mg/g (303 K), and 35.92 mg/g (313 K) (Figure 6H; Table 3). These results significantly validate the remarkable increment in the quantities of the existing sites after the integration between PANI and ZA. This was attributed strongly to the associated increase in the surface area and the incorporation of additional active functional sites after the formation of the composite, which induced the interactive interface between the dye solutions and the surface of the studied composite. Moreover, the present active sites on the surface of both ZA and PANI/ZA are of high selectivity and affinity towards basic MB dye as compared to acidic CR dye, which is reversible with the reported capacity of each site. 

The Nm _(MB)_ and Nm _(CR)_ values for both ZA and PANI/ZA possess reversible relationships with temperature in terms of how they are impacted by temperature (Figure 6E–H; Table 3). This is consistent with the values of n _(MB)_ and n _(CR)_ that have been previously observed because a rise in aggregation affinity decreases the number of occupied sites in addition to the effect of temperature on the dominant status of the present active sites [63,64,65]. The high-temperature settings have a beneficial activating effect on the ZA and PANI/ZA uptake sites, either by exposing extra active sites or additional energetic site groups [64]. The rise in adsorption temperature correlates with a reduction in the degree of viscosity of the MB and CR solutions [66]. As a result, the water-soluble MB and CR molecules show a substantial rise in their mobility as well as their diffusion behaviour when in contact with more active sites.

Adsorption capacity at the saturation state of (Qsat)

The MB and CR adsorption performances of ZA and PANI/ZA at saturation (Q_sat_) indicate the most acceptable predicted value for the maximal uptake capacities. The values of Q_sat_ are influenced by two critical variables: the recognized density of the occupied sites (Nm) as well as the total number of captured dye molecules by each site (n). As a potential adsorbent of MB, ZA has computed Q_sat_ values of 179.6 mg/g (293 K), 145.11 mg/g (303 K), and 115.6 mg/g (313 K) (Figure 6I; Table 3). The application of it during the decolourization of CR exhibits predicted saturation capacities of 140.3 mg/g (293 K), 110.04 mg/g (303 K), and 89.6 mg/g (313 K) (Figure 6J; Table 3). Regarding the application of the ZPANI/ZA composite, potential enhanced uptake capacities are achieved at its saturation states, either during the uptake of the MB (270.9 mg/g (293 K), 218.2 mg/g (303 K), and 171.4 mg/g (313 K)) or the CR dye (235.5 mg/g (293 K), 185.5 mg/g (303 K), and 158.2 mg/g (313 K)) (Figure 6K,L; Table 3). The exothermic features of the MB and CR uptake processes by ZA and PANI/ZA are influenced by the negative influence of temperature (Figure 6I–L; Table 3). This also indicated the accelerating influence of the uptake temperature on thermal collisions, which produces a decrease in the effectiveness of the MB and CR adsorption capacity [63]. Furthermore, the obtained characteristics of Q_sat_ as a consequence of uptake temperature reflect agreement with the previously described behaviours of Nm as opposed to n, suggesting the regulating impact of the existing active sites on the effectiveness of the adsorption instead of the capacity of each active site.

##### Energetic Properties

Adsorption energy

The energies (ΔE) of the MB and CR uptake reactions may consistently demonstrate the type of influencing mechanisms, whether they are chemical or physical procedures. While the influencing physical processes have energy values of less than 40 kJ/mol, the chemical mechanisms have energy levels greater than 80 kJ/mol. According to the adsorption energy values, the physically occurring mechanistic processes are also divided into various categories. These include coordination exchange (40 kJ/mol), dipole bonding forces (2–29 kJ/mol), hydrogen binding (<30 kJ/mol), the forces of van der Waals (4–10 kJ/mol), and hydrophobic bonding (5 kJ/mol) [47]. The values of the MB and CR capturing energies (ΔE) were calculated mathematically using Equation (5) using the obtained solubility values of MB and CR separately in the aqueous solution (*S*), gas constant (R = 0.008314 kJ/mol.K), MB and CR concentrations at the half saturation conditions of ZA and PANI/ZA, and absolute temperature (T) [65].

(1) $$∆E=RT\;ln\left(\frac{S}{C}\right)$$

The energy estimates for MB and CR sequestration by ZA are within the range of −10.26 to −16.8 kJ/mol and −9.38 to −16.49 kJ/mol, respectively (Table 3), while the estimated values for PANI/ZA varied from −7.5 to −14.11 kJ/mol for the MB and from −8.94 to −12 kJ/mol for the CR dye (Table 3). Consequently, the physical mechanistic processes such as the van der Waals forces (4 to 10 kJ/mol), dipole bonding forces (2 to 29 kJ/mol), and hydrogen bonding (<30 kJ/mol) were mostly responsible for the uptake of both MB and CR by either ZA or PANI/ZA. Additionally, the strong negative indications of the calculated values of E during the capture of MB and CR, by either ZA or PANI/ZA, reflect the earlier presented findings from experiments about the exothermic characteristics of the occurring processes.

Thermodynamic functions
Entropy

The entropy (Sa) of the MB and CR uptake activities by ZA and PANI/ZA strongly illustrates the order and disorder features of the surfaces of its nanoparticles under the influence of various concentrations of the dye ions, besides the selected temperature of the evaluated reaction. The Sa behaviours have been displayed using the findings of Equation (6) using the previously reported values for Nm and n as well as the estimated MB and CR concentrations during the half-saturation stage of ZA and PANI/ZA (C1/2).

(2) $$\frac{S_{a}}{K_{B}}=Nm\left\{\ln\left(1+{\left(\frac{C}{C_{\frac{1}{2}}}\right)}^{n}\right)-n{\left(\frac{C}{C_{\frac{1}{2}}}\right)}^{n}\;\frac{ln\left(\frac{C}{C_{\frac{1}{2}}}\right)}{1+{\left(\frac{C}{C_{\frac{1}{2}}}\right)}^{n\;}}\;\right\}$$

**Figure 7:**
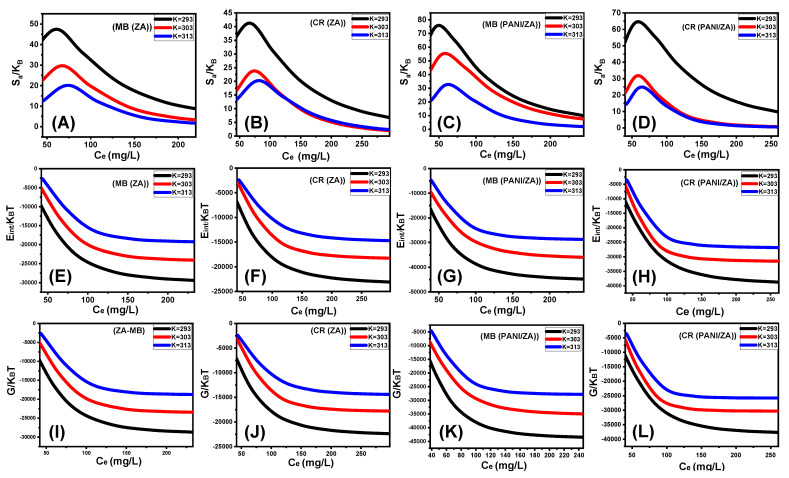
The changes in entropy during the adsorption of the dyes by ZA (MB (**A**) and CR (**B**)) and PANI/ZA (MB (**C**) and CR (**D**)), changes in the internal energy during the uptakes of the dyes by (MB (**E**) and CR (**F**)) and PANI/ZA (MB (**G**) and CR (**H**)), and changes in the enthalpy during the adsorption of dyes by ZA (MB (**I**) and CR (**J**)) and PANI/ZA (MB (**K**) and CR (**L**).

According to the observed curves, the levels of the entropy (Sa) values throughout the uptake of MB and CR, by either ZA (Figure 7A,B) and PANI/ZA (Figure 7C,D), fall significantly for the tests performed in the presence of extremely high MB and CR concentrations. This trend reveals the dramatic decrease in the disorder characteristics of the surfaces of both ZA and PANI/ZA when increasing the tested MB and CR concentrations. These entropy characteristics additionally endorse the successful docking of the MB and CR within the ZA and PANI/ZA existing active and free binding receptors in the presence of low levels of the dye ions [65,66]. Entropy maxima were recorded during the capture of MB by ZA at equilibrium concentrations of 74.16 mg/L (293 K), 67.8 mg/L (303 K), and 61.08 mg/L (313 K). Regarding the corresponding equilibrium concentrations of maximum entropy during the uptake of CR by ZA, the determined values are 74.16 mg/L (293 K), 74.6 mg/L (303 K), and 80.5 mg/L (313 K). For the application of PANI/ZA as an adsorbent for MB, the identified maxima values of entropy correspond to 62.6 mg/L (293 K), 56.5 mg/L (303 K), and 47.7 mg/L (313 K). The reported values during its application as an adsorbent for CR are 64.5 mg/L (293 K), 59.3 mg/L (303 K), and 55.8 mg/L (313 K). These equilibrium levels are quite similar to those predicted for the concentrations of the two dyes during the half-saturation stages of ZA and PANI/ZA. As a result, extra MB and CR ions are unable to dock onto the free sites of them. Additionally, the striking decreases in the measured values of entropy pointed to a sharp loss in the quantity of free sites and a considerable reduction in the MB and CR ions’ freedom and diffusion characteristics [66].

Internal energy and free enthalpy

The internal energy (E_int_) corresponding to the MB and CR uptake activities by ZA and PANI/ZA together with the free enthalpy (G) characteristics and how they change as a consequence of variations in the dye concentrations and operating temperature were evaluated utilizing the values derived from Equations (7) and (8), respectively, based on the previously determined Nm, *n*, and C_1/2_ in addition to the translation partition (*Z_v_*) [63].

(3) $$\frac{E_{int}}{K_{B}T\;}=n\;N_{m}\left[\left(\;\frac{{\left(\frac{C}{C_{1/2}}\right)}^{n}\;ln\left(\frac{C}{Z_{v}}\right)}{1+{\left(\frac{C}{C_{1/2}}\right)}^{n}}\right)-\left(\;\frac{n\ln\left(\frac{C}{C_{1/2}}\right)\;{\left(\frac{C}{C_{1/2}}\right)}^{n}}{1+{\left(\frac{C}{C_{1/2}}\right)}^{n}}\right)\right]$$

(4) $$\frac{G}{K_{B}T\;}=n\;N_{m}\frac{\ln\left(\frac{C}{Z_{v}}\right)}{1+{\left(\frac{C_{1/2}}{C}\right)}^{n}}$$

The derived E_int_ values for the performed MB and CR uptake activities by ZA (Figure 7E,F) and PANI/ZA (Figure 7G,H) have negative signs, and these results exhibit a striking decline whenever the investigated temperature is raised from 293 K to 303 K. This reinforces the spontaneous and exothermic properties of the MB as well as the CR uptake reactions by both ZA and PANI/ZA. The same responses and characteristics were observed for the established enthalpy levels. The G values are negatively signed and exhibit a reversible relationship with the experimental uptake temperature, indicating a decline in the feasibility characteristics and confirming the spontaneous and exothermic behaviour of the MB and CR uptake by both ZA (Figure 7I,J) and PANI/ZA composite (Figure 7K,L).

## 3. Materials and Methods

### 3.1. Materials

Kaolinite, which was used as a precursor during the production of zeolite, was delivered from the Central Metallurgical Research and Development Institute in Egypt. Aniline monomer was obtained (Rankem Company, Delhi, India), and (NH_4_)_2_S_2_O_8_ (Win Lab Corporation, London, UK) was applied during the synthesis of polyaniline. NaOH (Sigma-Aldrich, Cairo, Egypt), HNO_3_ (Sigma-Aldrich, Egypt), and HCl (Sigma-Aldrich, Egypt) were used during the different preparation and adjustment steps without purification. CR and MB synthetic dyes (Sigma-Aldrich, Egypt) were used as sources of the pollutants during the adsorption test.

### 3.2. Synthesis of the PANI/Zeolite-A Composite

#### 3.2.1. Synthesis of Zeolite-A

The synthesis of zeolite-A was accomplished considering the reported procedures [47]. Firstly, the kaolinite powder was thermally transformed into reactive metakaolinite via calcination for 4 h at 750 °C. After that, the meta-kaolinite powder was homogenized within the prepared NaOH solution (1.5 M; 100 mL) at an adjusted weight ratio of 1 (meta-kaolinite): 2 (NaOH) for 12 h using a magnetic stirrer at 500 rpm. After that, the mixture was carefully transferred into a hydrothermal alteration system composed of a Teflon-lined stainless-steel autoclave and treated thermally for four hours at 150 °C. The synthetic zeolite was separated through filtration and washed extensively with distilled water to remove the excess NaOH and neutralize the zeolite surface. Then, the zeolite particles were dried at 70 °C for 12 h and kept being incorporated in the next synthesis steps.

#### 3.2.2. Synthesis of Polyaniline/Zeolite-A Composite (PANI/ZA)

The sudden in situ polymerization of aniline prepared the composite in the presence of the zeolite grains. One gram of zeolite was dispersed within 50 mL of distilled water using a magnetic stirrer for four hours. The obtained suspension was mixed with a pre-prepared solution of 1 M aniline (0.46 mL) dissolved in 0.5 M HCL (2.5 mL) in the presence of a sonication source. After 20 min, 0.15 M of (NH_4_)_2_S_2_O_8_ dissolved in HCL (0.5 M; 50 mL) was added to the aniline/zeolite mixture and left for a certain interval under stirring to complete the polymerization of polyaniline over the surface of the zeolite grains. By the end of this step, the obtained solid products were isolated via centrifugation, washed several times with distilled water, dried at 60 °C for five hours, and labelled as PANI/ZA.

### 3.3. Adsorption Studies

#### 3.3.1. The Batch Adsorption Tests

The adsorption properties of PANI/ZA as an adsorbent of CR and MB dyes in comparison with synthetic ZA particles were studied according to a series of batch uptake tests, considering affecting variables such as pH (3 to 8), the prepared concentration of dyes (50 to 400 mg/L), and adsorption duration (30 to 1440 min). The volumes of the dyes and the used dosage of PANI/ZA were fixed at 100 mL and 0.2 g/L, respectively, while the adsorption temperature was studied within an experimental interval from 303 K to 323 K. The adsorption results were evaluated at their average values, considering the performance of the experiment in triplicate. By the end of each test, the rest of the dyes were measured using a UV–Vis spectrophotometer after adjusting the maximum wavelength at 450 nm and 430 nm for the CR dye and MB dye, respectively. The determined concentration of the dyes was used to calculate the adsorption capacity of PANI/ZA according to Equation (1), considering also the treated volume (V), the composite dosage (m), the initial concentration (*C_o_*), and the rest concentration (*C_e_*).

(5) $$Q_{e(mg/g)}=\frac{\left(C_{O}-C_{e}\right)V}{m}$$

#### 3.3.2. Theoretical Traditional and Advanced Equilibrium Studies

The adsorption experiments were theoretically modelled using classical kinetic, classic isotherm, and advanced isotherm modelling based on statistical physics hypotheses (Table 4). The kinetic and classic equilibrium modelling were conducted using nonlinear fitting procedures, utilizing the theoretical formulas of these models and the resultant values of the correlation coefficient (R^2^) (Equation (2)) in addition to Chi-squared (χ^2^) (Equation (3)). The fitting levels of the adsorption processes with the evaluated advanced equilibrium models were determined using the correlation coefficient (R^2^) and the root mean square error (RMSE) (Equation (4)). The m′, p, Qi_cal_, and Qi_exp_ symbols represent the obtained experimental results, investigated factors, the assumed CR and MB adsorption, and the validated adsorption capacity, respectively.

(6) $$R^{2}=1-\frac{\sum (q_{e,\;exp}-q_{e,\;cal}{)}^{2}}{\sum (q_{e,\;exp}-q_{e,\;mean}{)}^{2}}$$

(7) $$\chi^{2}=\sum \frac{(q_{e,exp}-q_{e,cal}{)}^{2}}{q_{e,cal}}$$

(8) $$RMSE=\sqrt{\frac{\sum_{i=1}^{m}({Qi}_{cal}-{Qi}_{exp}{)}^{2}}{m^{\prime }-p}}$$

## 4. Conclusions

The adsorption performances of a synthetic PANI/ZA composite as an adsorbent for MB and CR dyes have been studied, considering the synergetic enhancement impact of the PANI polymers on their uptake capacities. The PANI/Za composite displayed significantly enhanced uptake properties ((270.9 mg/g) and CR (235.5 mg/g)) as compared to ZA (MG (179.6 mg/g) and CR (140.3 mg/g)). The MB and CR adsorption behaviours by PANI/ZA, as well as the affecting mechanisms, were evaluated according to the isotherm assumptions of the conventional Langmuir model as well as an advanced monolayer model with one site of energy. The steric studies demonstrated a remarkable increase in the existing quantities of the effective uptake sites after the incorporation of PANI (MG (109.2 mg/g) and CR (92.9 mg/g)). This explains the significantly higher MG and CR uptake capacities of PANI/ZA than ZA. Furthermore, the estimated number of adsorbed MG molecules (up to four molecules) and Cr molecules (up to five molecules) declared their uptake to be through multi-molecular mechanisms in vertical ordering. The determined uptake energies of MG and CR (<40 kJ/mol), as well as the thermodynamic functions, declared their uptake to be through exothermic, physical, and spontaneous mechanisms.

## Figures and Tables

**Table 1:** The mathematical parameters of the addressed kinetic models.

Kinetic Models					
Models	Parameters	ZA (MB)	ZA (CR)	PANI/ZA (MB)	PANI/ZA (CR)
Pseudo-first-order	*k*_1_ (1/min)	0.0116	0.0095	0.0125	0.0121
	Qe _(Cal)_ (mg/g)	111.5	83.2	130.5	112.8
	R^2^	0.97	0.926	0.985	0.95
	χ^2^	0.594	2.25	0.372	1.039
Pseudo-second-order	*k*_2_ (mg/g min)	9.67 × 10^−5^	9.14 × 10^−5^	9.28 × 10^−5^	1.31 × 10^−4^
	Qe _(Cal)_ (mg/g)	129.75	100.27	150.5	114.8
	R^2^	0.937	0.89	0.96	0.92
	χ^2^	1.40	3.26	1.03	1.94

**Table 2:** The estimated mathematical parameters of the studied classic and advanced equilibrium models.

Parameters of the Classic Isotherm Models													
Models	Parameters	MB (ZA)			CR (ZA)			MB (PANI/ZA)			CR (PANI/ZA)		
		293 K	303 K	313 K	293 K	303 K	313 K	293 K	303 K	313 K	293 K	303 K	313 K
Langmuir	Q_max_ (mg/g)	179.65	145.11	115.6	140.36	110.04	89.6	270.9	218.02	177.4	235.5	189.8	166.4
	b(L/mg)	3.14 × 10^−5^	1.03 × 10^−6^	6.06 × 10^−8^	8.06 × 10^−5^	1.41 × 10^−6^	1.91 × 10^−6^	6.41 × 10^−5^	1.54 × 10^−5^	3.12 × 10^−7^	3.41 × 10^−5^	7.3 × 10^−8^	1.14 × 10^−8^
	R^2^	0.994	0.99	0.991	0.99	0.994	0.991	0.996	0.993	0.994	0.998	0.996	0.994
	χ^2^	0.22	0.102	0.048	0.048	0.068	0.013	0.0085	0.030	0.269	0.125	0.118	0.243
Freundlich	1/n	0.53	0.43	0.38	0.56	0.44	0.47	0.55	0.50	0.39	0.55	0.35	0.33
	k_F_ (mg/g)	189.01	151.3	120.3	146.4	114.6	94.04	285.6	228.9	177.4	249.7	190.9	163.3
	R^2^	0.98	0.974	0.983	0.987	0.991	0.974	0.982	0.991	0.983	0.99	0.98	0.986
	χ^2^	0.654	0.836	0.554	0.461	0.124	0.257	0.452	0.234	0.314	0.413	0.568	0.539
D-R	β (mol^2^/KJ^2^)	0.00891	0.0129	0.0181	0.00794	0.0164	0.0213	0.00560	0.00839	0.0119	0.00785	0.00866	0.0127
	Q_m_ (mg/g)	178.3	153.8	127.4	136.58	115.04	92.46	266.2	219.5	184.5	231.3	203.2	176.8
	R^2^	0.98	0.99	0.99	0.988	0.999	0.996	0.99	0.99	0.99	0.986	0.992	0.987
	χ^2^	0.57	0.24	0.34	0.353	0.0171	0.092	0.64	0.235	0.176	0.861	0.537	0.713
	E (KJ/mol)	7.49	6.22	5.25	7.93	5.52	4.84	9.44	7.71	6.48	7.98	7.59	6.27

**Table 3:** The mathematical parameters of the studied advanced isotherm model.

Advanced Isotherm Model				
Steric and Energetic Parameters				
		293 K	303 K	313 K
MB (ZA)	n	2.58	3.34	3.92
	Nm (mg/g)	69.39	43.45	29.45
	Q_Sat_ (mg/g)	179.6	145.1	115.6
	C1/2 (mg/L)	54.85	62.06	68.9
	ΔE (kJ/mol)	−10.26	−13.72	−16.8
CR (ZA)	n	2.31	3.045	3.17
	N_m_ (mg/g)	60.67	34.66	29.43
	Q_Sat_ (mg/g)	140.3	110.04	89.6
	C1/2 (mg/L)	58.8	69.57	75.38
	ΔE (kJ/mol)	−9.38	−13.9	−16.49
MB (PANI/ZA)	n	2.48	2.73	3.65
	N_m_ (mg/g)	109.2	79.7	47.5
	Q_Sat_ (mg/g)	270.9	218.2	173.4
	C1/2 (mg/L)	48.99	57.47	61.92
	ΔE (kJ/mol)	−7.5	−11.7	−14.11
CR (PANI/ZA)	n	2.53	4.08	4.5
	N_m_ (mg/g)	92.9	45.93	35.92
	Q_Sat_ (mg/g)	235.5	187.4	161.7
	C1/2 (mg/L)	57.74	57.97	63.54
	ΔE (kJ/mol)	−8.94	−9.35	−12.0

**Table 4:** Nonlinear equations of kinetic, classic isotherm, and advanced isotherm models.

Kinetic Models		
Model	Equation	Parameters
Pseudo-first-order	$Q_{t\;}=Q_{e}\;(1-e^{{-k}_{1}t})$	*Q*_t_ (mg/g) is the adsorbed ions at time (t), and *k*_1_ is the rate constant of the first-order adsorption (1/min)
Pseudo-second-order	$Q_{t}=\frac{Q_{e\;}^{2}k_{2}t}{1+Q_{e}k_{2}t}$	Q_e_ is the quantity of adsorbed ions after equilibration (mg/g), and *k*_2_ is the model rate constant (g/mg min)
Classic Isotherm models		
Model	Equation	Parameters
Langmuir	$Q_{e}=\frac{Q_{max}\;bC_{e}}{\left(1+bC_{e}\right)}$	*C_e_* is the rest concentration (mg/L), Q_max_ is the theoretical maximum adsorption capacity (mg/g), and *b* is the Langmuir constant (L/mg)
Freundlich	$Q_{e}=K_{f}C_{e}^{1/n}$	*K_f_* (mg/g) is the constant of the Freundlich model related to the adsorption capacity, and n is the constant of the Freundlich model related to the adsorption intensities
Dubinin–Radushkevich	$Q_{e}=Q_{m}e^{-\beta \varepsilon^{2}}$	*β* (mol^2^/KJ^2^) is the D-R constant, *ε* (KJ^2^/mol^2^) is the polanyil potential, and *Q_m_* is the adsorption capacity (mg/g)
Advanced isotherm models		
Model	Equation	Parameters
Monolayer model with one energy site (Model 1)	$Q=nN_{o}=\frac{nN_{M}}{1+{\left(\frac{C1/2}{C}\right)}^{n}}=\frac{Q_{o}}{1+{\left(\frac{C1/2}{C}\right)}^{n}}$	Q is the adsorbed quantity in mg/g *n* is the number of adsorbed ions per site Nm is the density of the effective receptor sites (mg/g) *Q_o_* is the adsorption capacity at the saturation state in mg/g C_1/2_ is the concentration of the ions at half saturation stage in mg/L C_1_ and C_2_ are the concentrations of the ions at the half saturation stage for the first active sites and the second active sites, respectively *n_1_* and *n_2_* are the adsorbed ions per site for the first active sites and the second active sites, respectively
Monolayer model with two energy sites (Model 2)	$Q=\frac{n_{1}N_{1M}}{1+{\left(\frac{C_{1}}{C}\right)}^{n_{1}}}+\frac{n_{2}N_{2M}}{1+{\left(\frac{C_{2}}{C}\right)}^{n_{2}}}$	
Double layer model with one energy site (Model 3)	$Q=Q_{o}\frac{({\frac{C}{C1/2})}^{n}+2({\frac{C}{C1/2})}^{2n}}{1+({\frac{C}{C1/2})}^{n}+({\frac{C}{C1/2})}^{2n}}$	
Double layer model with two energy sites (Model 3)	$Q=Q_{o}\frac{({\frac{C}{C1})}^{n}+2({\frac{C}{C2})}^{2n}}{1+({\frac{C}{C1})}^{n}+({\frac{C}{C2})}^{2n}}$	

## Data Availability

Data are available upon reasonable, by the Corresponding Authors.
